# Investigation of the relationship between organizational cynicism and counterproductive work behaviors: a systematic review and meta-analysis

**DOI:** 10.3389/fpsyg.2025.1529798

**Published:** 2025-11-26

**Authors:** Aydın Çivilidağ, Şerife Durmaz, Berk Uslu

**Affiliations:** 1 Department of Psychology, Faculty of Literary, Akdeniz University, Antalya, Türkiye; 2 Department of Labor Economics and Industrial Relations, Faculty of Economics and Administrative Sciences, Akdeniz University, Antalya, Türkiye; 3 Department of Labor Economics and Industrial Relations, Institute of Social Sciences, Akdeniz University, Antalya, Türkiye

**Keywords:** work life, organizational behaviors, organizational cynicism, counterproductive work behaviors, meta-analysis

## Abstract

**Introduction:**

The aim of the research is to examine the effect size between organizational cynicism and counterproductive work behaviors, which are within the scope of negative employee attitudes and behaviors in working life, with the systematic review method.

**Methods:**

The systematic review was conducted in accordance with PRISMA guidelines. EBSCO, Google Scholar, SAGE, Scopus, WILEY and WoS databases were searched by the authors with the keywords “Organizational cynicism, employee cynicism, deviant work behaviors, workplace deviant behavior,” “Counterproductive work behaviors,” “employee’s misbehavior” in English between 2010 and 2024.

**Results:**

The data set of the research consists of 22 empirical studies conducted in 9 different countries with 7,331 participants. According to the random effects model, the effect size was found 0.482 between organizational cynicism and counterproductive work behaviors. According to this result, a medium and significant effect size and a positive relationship (*r* = 0.448) between organizational cynicism and counterproductive work behaviors were found.

**Discussion:**

Organizations should consider that each employee is negatively affected by negative leadership behaviors, unfair and unequal organizational decisions and practices. Therefore, it can be said that equal, fair, supportive attitudes and organizational practices will reduce employees’ organizational cynicism and counterproductive work behaviors and also positively affect their organizational commitment, job performance and organizational citizenship behaviors.

## 1 Introduction

In today’s business life, organizations employ employees who have different expertise, knowledge, skills and abilities to achieve their organizational goals. Organizations place their members in different positions, tasks and responsibilities at different levels of a hierarchical structure to achieve a collective set of goals and objectives. In this respect, organizations are social systems that require interaction among people (Nassehi, 2005). While creating a balance of authority and responsibility between the abilities of employees and the requirements of the job, the organization considers the personalities, wishes and goals of them (Sabuncuoğlu and Vergiliel Tüz, 2016). Therefore, irrational decisions and practices taken by organizations in some cases can negatively affect employees’ feelings, thoughts, behaviors and attitudes (Nassehi, 2005). In addition to employees’ perceptions of organizational injustice, low organizational trust and low organizational support, various situations such as unethical leadership behaviors of managers and mobbing of employees have been identified as the causes of organizational cynicism (FitzGerald, 2002; Bedük et al., 2017; Akar, 2019; Hussain et al., 2021; Yalçın and Özbaş, 2021). According to Chiaburu et al. (2013), if employees feel that their contracts have been violated, they may think that the organization is not being honest with them. This is a violation of the psychological contract and can lead to a lack of trust to the organization. Role ambiguity in the workplace and the inability to fulfill family responsibilities due to work-related reasons can cause psychological tension and work stress in employees. All these negative workplace experiences can lead to organizational cynicism in employees. Just as there may be cynical individuals in the society for various reasons (injustice, violation of personal rights, etc.), it is also possible to encounter cynical employees in business life. In addition to damaging behaviors such as revenge and retaliation, employees may engage in illegal behavior against the organization or its employees when they feel they are being put in a difficult position by the organization (Spector, 2011; Roxana, 2013; Oliveira et al., 2020). For whatever reason, both organizational cynicism and counterproductive work behaviors cause important economic, psychological and social damage to both the organization and employees (Shahzad and Mahmood, 2012; Robinson and Bennett, 1995; Rayan et al., 2018). The Hawthorne studies of Mayo and his colleagues, which paved the way for the human relations movement in the field of organizational behavior, revealed that organizations do not consist of mechanical systems and drew attention to human capital as the most important element of production (Muldoon, 2017). The Hawthorne studies temporarily shifted academic attention away from the physical work environment and stimulated a literature examining rich human interactions within organizations. Thus, it set the stage for future research into the complex relationships between social dynamics and the physical work environment (Zhong and House, 2012). The psychological, social and cultural structure of human beings is both affects and affected by the organization that constitutes a social system. In addition, changing socioeconomic life, technology, culture of employee, attitudes and behaviors of managers and business life affect employees and organizations. Cultural values can significantly affect employees’ attitudes and feelings toward the organization. Hofstede’s (2011) cultural dimensions (power distance, individualism/collectivism, uncertainty avoidance, etc.) have significant relationships with organizational commitment, citizenship behavior and emotional reactions. These relationships may also extend to negative attitudes such as organizational cynicism. Individualism/collectivism dimension is one of the most determinant cultural factors in organizational behavior differences. This dimension can play an important role in the attitudes and behaviors (e.g., cynicism) that employees develop toward the organization (Taras et al., 2010). There are many studies on organizational cynicism and counterproductive work behaviors in the literature. However, there is no meta-analysis study synthesizing these studies. Therefore, it is important to determine the effect size between organizational cynicism and counterproductive work behaviors and to reveal the relationship between these two phenomena. Each empirical research has a unique nature. For example, even if there are studies on the same subject, different measurement tools, participants with characteristics from different cultures, subcultures even within the same culture, and different organizational cultures can be listed as factors that may affect the studies. Therefore, the meta-analysis method allows synthesizing such differences and reaching inductive results. The purpose of this study is to examine the relationship between organizational cynicism and counterproductive work behaviors through systematic review method. In line with the theoretical framework of the research, organizational cynicism and counterproductive work behaviors are explained below.

### 1.1 Theoretical background

#### 1.1.1 Organizational cynicism

According to Andersson and Bateman (1997), cynicism can be directed toward a specific object or multiple objects. Cynicism can be defined as both a general and specific attitude characterized by negative feelings, disappointment and distrust toward a person, group, ideology, social custom or institution. Organizational cynicism occurs when employees do not trust their organizations and feel that the organization cannot be trusted (Durrah et al., 2019). If employees feel distrust and injustice toward their organizations, they may change their thoughts, attitudes and behaviors toward the organization and their colleagues. Organizational cynicism is a negative attitude of employees toward their organizations and consists of three dimensions: (1) belief that the organization is dishonest; (2) negative affectivity toward the organization; (3) dismissive and critical behavioral tendencies toward the organization consistent with these beliefs and affect. Cynicism is not a neutral judgment about the organization; it may involve strong emotional reactions (Dean et al., 1998). According to Abraham (2000), organizational cynicism is the negative feelings of employees toward the organization due to the attitudes and behaviors of leaders or managers in cynicism, employees’ negative feelings toward the organization are based on a strong discredit of honesty, fairness and sincerity. The emotional component of cynicism refers to the arousal of strong negative emotions such as humiliation, anger, discomfort and shame. The occurrence of organizational cynicism is based on the belief that the organization lacks honesty. In other words, employees’ perceptions or experiences of dishonest or unfair behavior, insincerity, and their beliefs about the organization can lead to organizational cynicism (Naus et al., 2007). Social exchange theory explains the distrustful, pessimistic (cynical) behavior of people working in an organization. Social Exchange Theory explains that the fundamental nature of human behavior is a subjective interaction with others. It emphasizes that the development of interpersonal relationships is based on the norm of reciprocity (Jiang et al., 2017). Social exchange theory is examined as the conceptual basis of organizational cynicism and based on the argument that organizational cynicism is a result of psychological contract breach (Pfrombeck et al., 2020). Johnson and O’Leary-Kelly (2003) determined that cynicism partially mediates the effects of psychological contract breach on work-related attitudes. McMillan and Albrecht (2010) also found a significant relationship between cynicism and social exchange and emotional commitment. While employees approach the organization with attitudes such as loyalty, labor and commitment, they expect fair treatment, trust and appreciation from the organization in return. When these expectations are not met by the organization, they lose their belief in the sincerity of the organization and develop cynical attitudes (Chiaburu et al., 2013). Therefore, social exchange theory reveals that employees cognitively evaluate organizational practices and attitudes toward themselves in the organizational structure. When employees perceive that they are treated unkindly and badly in the workplace for any reason, they may develop cynical attitudes toward the organization in return. Another approach that explains organizational cynicism behaviors is the job demands-resources (Job demands-R) model. According to Demerouti et al. (2001), job demands are “the physical, psychological, social, or organizational aspects of work that require sustained physical or mental effort and are therefore associated with certain physiological and psychological costs (e.g., stress and exhaustion).” As work demands increase, they can lead to fatigue and health problems in employees. If job demands, such as high work pressure and violent conflicts persist over time, employees’ relationships may deteriorate, health problems may become chronic and work performance may decline. On the other hand, job demands and resources are the initiators of the motivation process. Therefore, they are the most important determinants of employees’ job commitment and organizational commitment (Bakker, 2015). Job resources refer to the physical, psychological, social or organizational aspects of work that promote personal growth, learning and development that are functional for achieving work goals. Autonomy, skill diversity, performance feedback and growth opportunities are examples of job resources (Bakker and Demerouti, 2016). The JD-R model tries to explain two causal processes: the health impairment process and the motivational process. High job demands (such as workload, time pressure, emotional demands) increase the risk of burnout in employees and can lead to negative outcomes such as health complaints or turnover intentions. Job resources (autonomy, social support, performance feedback, and professional development opportunities) play a motivational role in employees, fostering commitment to work and promoting positive organizational outcomes such as performance or organizational commitment. Moreover, while the absence of job resources leads to burnout, job resources can buffer the impact of job demands on burnout (Lesener et al., 2019). While the intensity of job demands can lead to stress, tension, burnout and cynical feelings toward the organization, job resources have the effect of reducing the psychological and physiological costs caused by job demands. Employees with negative attitudes and feelings toward the organization may indicate counterproductive work behaviors in order to cope with this situation or to achieve justice for themselves. Social exchange theory defines relationships between people as interactions in which people have obligations toward each other. Therefore, when employees see positive (or negative) attitudes and behaviors toward them in their organizations, they feel an obligation to show positive (or negative) attitudes and behaviors in return (Evans et al., 2021). Studies examining the relationship between organizational cynicism and counterproductive work behaviors have revealed a significant and positive relationship between these two phenomena (Evans et al., 2010; Ahmed et al., 2013; Shahzad and Mahmood, 2012; Rayan et al., 2018; Ali et al., 2020; Dar et al., 2020).

#### 1.1.2 Counterproductive work behaviors

Counterproductive work behaviors are generally defined as deviant behaviors in the workplace that are contrary to organizational norms. According to Marcus and Schuler (2004), these behaviors include theft, fraud, absenteeism, physical and verbal aggression or drug addiction. Deviant behaviors in the workplace are unethical actions of individuals or groups whose attitudes and actions differ from accepted social standards (Lugosi, 2019). According to Spector and Fox (2002), deviant workplace behaviors are negative and harmful behaviors that violate organizational norms. For example, absenteeism, not doing work properly, physical anger, verbal hostility (humiliation), sabotage and theft. While anger and hostility are directly individual-oriented, not doing things properly or sabotage are organization-oriented. Theft is both individual and organizational oriented behavior. According to Robinson and Bennett (1995), counterproductive work behaviors are taxonomically divided into two groups: organizational and individual behaviors. First, organizational behaviors are minor organizational-oriented behaviors such as leaving work early, taking frequent breaks, deliberately slowing down work, and wasting resources. On the other hand, sabotage, taking bribes and stealing organizational property are classified as major organizational behaviors. Secondly, individual behaviors are those that are considered minor: interpersonal behaviors (favoritism, gossiping about co-workers, blaming co-workers) and major interpersonal behaviors (sexual harassment, verbal harassment, stealing co-workers’ belongings, endangering co-workers). In addition to individual reasons such as personality, genetic factors, family background and social influences, organizational and work-related reasons (perception of injustice, frustration, job satisfaction and work stress) are also suggested to lead employees to such behaviors (Marcus and Schuler, 2004; Agrawal and Pandey, 2021). It can be said that three models stand out in the studies on counterproductive work behaviors. (1) The model that Robinson and Bennett (1995) and Bennett and Robinson (2000) divided into two as organizational deviance (OD) and interpersonal deviance (ID). (2) Spector et al. (2006) five-dimensional model that includes abuse, production deviance, theft, sabotage, and withdrawal; and (3) the more elaborate 11-faceted model proposed by Gruys and Sackett (2003) that includes theft, property damage, information misuse, time and resource misuse, unsafe behaviors, absenteeism, poor work quality, alcohol use, drug use, inappropriate verbal behavior, and inappropriate physical behavior (Marcus et al., 2016). Regarding the antecedents of employees’ counterproductive work behaviors, Zappala et al. (2022) put forward three main ideas. The first main idea is the sociodemographic and personality aspects of perpetrators and victims. Secondly, the effects of organizational contexts on individual deviant behaviors and finally the role of the leader who influences the organizational climate. Hafidz (2012) stated that in general, antecedents of counterproductive work behaviors can be divided into individual (personality, locus of control and values) and situational factors. Ahmed et al. (2013) explained that counterproductive work behaviors are caused by many factors such as psychological contract breach, interactional injustice, cynicism, dissatisfaction, job autonomy, self-control and pay inequality. Bari et al. (2022) and Kobanoğlu and Erdoğan (2022) found that psychological contract breach perceived by employees has a significant positive effect on counterproductive work behaviors. They also found that organizational cynicism has a partial role between psychological contract breach and counterproductive work behaviors. According to social exchange theory, when employees feel exploited, devalued or treated unfairly in their relationships with the organization, they may engage in counterproductive work behaviors as retaliation (Chernyak-Hai and Rabenu, 2018; Kim et al., 2023). The equity theory developed by Adams and Freedman (1976) emphasizes that employees try to strike a balance between what they receive in return for their work activities and their work output. In doing so, employees make comparisons with their coworkers and peers. If what employees receive in return for their work activities is less than their peers, the perception of inequality emerges and employees indicate deliberate behaviors such as slowing down work by reducing work performance, absenteeism, making excuses for not going to work, extending meal breaks and break times at work to compensate for this. Literature on revenge has focused on deviant behaviors in the workplace (e.g., physical violence, bad-mouthing managers or coworkers). However, those who study revenge have also offered an alternative perspective on the genesis of these behaviors. Attention has been drawn to what can be considered the “functional” side of deviant work behaviors. A central assumption of the revenge literature is that these acts redress injustices and reorganize dysfunctional power relations in work organizations. Deviant behaviors in the workplace threaten organizational effectiveness, but the revenge literature suggests that the enactment of these behaviors can serve functional purposes for perpetrators and coworkers (Tepper and Henle, 2011). Although there have been many correlational studies between organizational cynicism and counterproductive work behaviors in the literature in the last 14 years, there is no meta-analysis study that synthesizes these studies. This is the most important reason for conducting this study. Thus, by calculating the effect size between organizational cynicism and counterproductive work behaviors, more effective prediction and analytical evaluations can be made. Therefore, the purpose of this study is to examine the effect size between counterproductive work behaviors and organizational cynicism by meta-analysis method based on the studies conducted between 2010 and 2024. Accordingly, the research question was formulated as follows.

1. What is the effect size and relationship between organizational cynicism and counterproductive work behaviors?

## 2 Materials and methods

**FIGURE 1:**
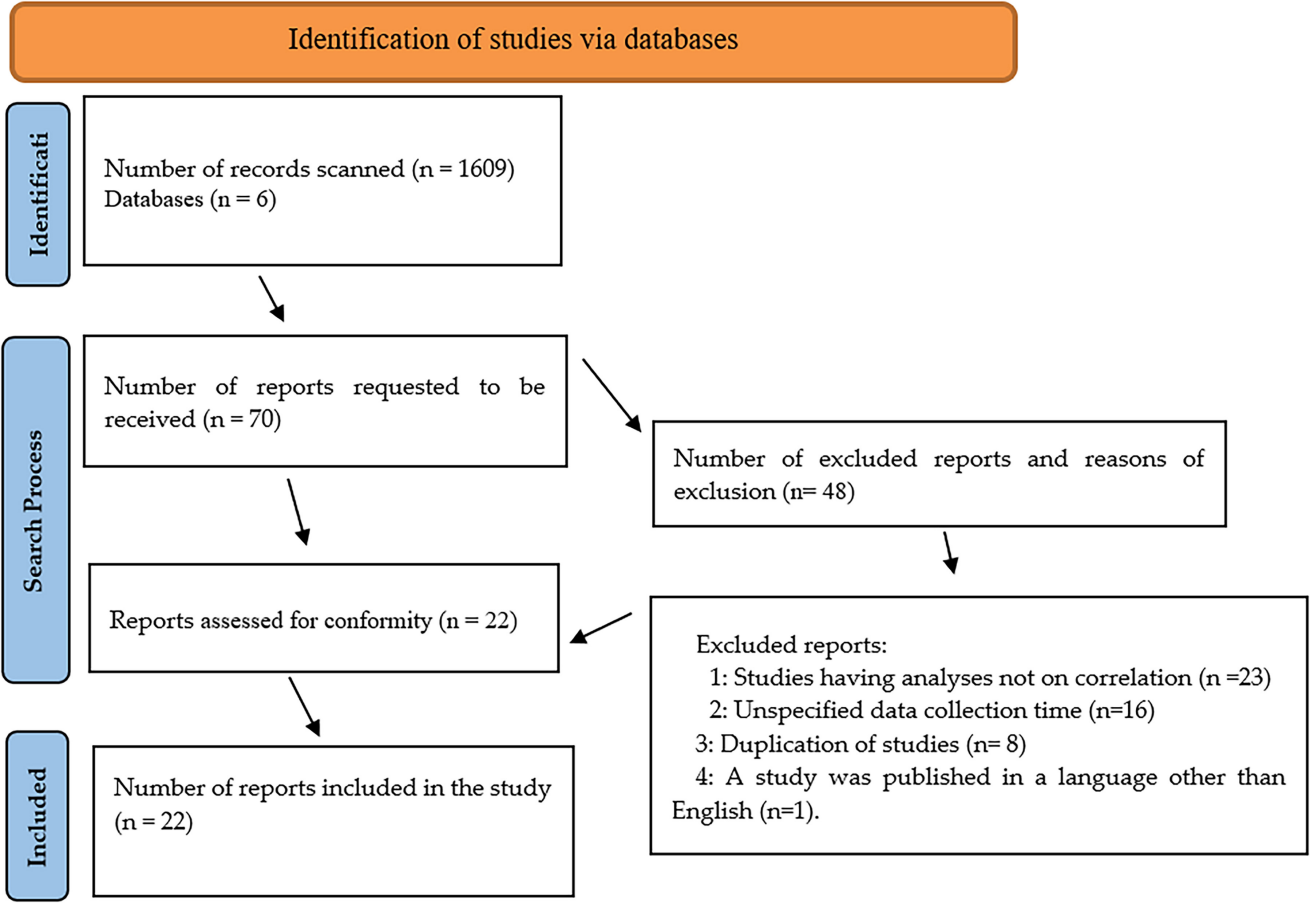
PRISMA flow diagram.

This research was conducted by using the systematic review method. The review was conducted in accordance with the PRISMA guidelines (Moher et al., 2009). Meta-analysis was applied to the studies included in the research. The research was conducted between January-September 2024 through EBSCO, Google Scholar, SAGE, Scopus, WILEY and WoS databases on Akdeniz University web page in line with the inclusion criteria determined jointly by three researchers. English keywords entered into the databases: “organizational cynicism, employee cynicism, deviant work behaviors, workplace deviant behaviors,” “counterproductive work behaviors,” “employee misbehavior,” “employee misbehavior in organizations” were searched with “AND, OR” Boolean operators. The researchers agreed on 6 databases that significantly cover studies in the field of social sciences. The researchers completed the search in 8 months by entering keywords and filtering the databases. The filtering was implemented in several steps. First, databases publishing research reports in the field of social sciences were selected and journals in the fields of organizational behavior, work psychology and business management were marked. In the first search, 1,609 studies were found. A total of 1,539 studies that did not meet the inclusion criteria were excluded by the consensus of the researchers. In the last stage, the researchers completed the screening process by integrating their data. Then, all researchers ensured rigor by checking the appropriateness of the studies they identified among themselves. While evaluating the quality of the studies, attention was considered whether the journals publishing the studies were peer-reviewed and indexed. The remaining 70 studies were entered into the Microsoft Excel program by all three researchers and read one by one. Of these studies, 22 studies that fully met the inclusion criteria were found eligible for the research with the approval of all researchers (Figure 1). The values of these 22 studies were entered into the CMA 3.0 program and analyzed for heterogeneity, effect size and publication bias and the data obtained are presented in the results section.

### 2.1 Eligibility criteria

The inclusion criteria were established by the researchers within a consensus framework. Accordingly, the main motivation for this research is the lack of a study that synthesizes the research on counterproductive work behaviors and organizational cynicism in the last 14 years and the abundance of correlative studies on these issues. In the last 10 years, epidemics and technological developments have significantly changed the working system and organizations. It is thought that these changes may also affect organizational behaviors. Therefore, it is aimed to examine the effect size and the relationship between organizational cynicism and counterproductive work behaviors. In this direction, the researchers included studies conducted between 2010 and 2024 and published electronically in social sciences databases in the inclusion criteria. Apart from these, all researchers accepted the other inclusion criteria stated below.

- Studies published electronically in journals in the field of social sciences between 2010 and 2024,

- Studies published in peer-reviewed and internationally indexed journals,

- Articles written in English,

- Studies examining organizational cynicism and counterproductive work behaviors,

- Studies that collect data using quantitative methods and surveys (face-to-face, e-mail or electronic surveys),

- Studies analyzed with Pearson correlation.

Exclusion Criteria: Unpublished articles, Master’s theses, dissertations, published or unpublished books, book chapters, conference or symposium proceedings are considered gray literature. Among these studies, theses, books and book chapters are excluded because they go through different evaluation and publication processes than articles. Conference and symposium papers are excluded because they are in progress and incomplete. Unpublished studies are excluded due to the possibility of retraction due to various potential errors (such as methodological, incorrect information, high similarity rates).

### 2.2 Selection and coding variables

The researchers independently read and analyzed the 70 studies that met the potential inclusion criteria and reported to be included in the research. The studies determined to be eligible for the research were independently and blindly entered Microsoft Excel spreadsheets by the researchers. Thus, the reliability of coding was ensured. The compatibility of the coders was checked by comparison, and disagreements were negotiated and corrected by consensus. The Excel spreadsheets were created by the researchers and then merged to a single form set. After the researchers confirmed that the studies met the specified criteria, meta-analysis was conducted on 22 studies. Table 1 shows the characteristics of the included studies (number of studies, authors, study title, quality score, study type, etc.).

**TABLE 1:** Checklist for the assessment of the methodological quality of the reviewed studies.

Checklist items	Points
**1. Sampling and representativeness (Sampling method)**	
Non-probability sampling (including purposive, quota, convenience and snowball sampling)	0
Probability sampling (including: simple random, systematic, stratified g, cluster, two-stage and multi-stage sampling)	1
**2. Are the individuals selected to participate in the study likely to be representative of the target population?**	
No	0
Yes	1
**3. Selection bias: Is there a risk of selection bias caused by the inadequate selection of participants**	
High risk	0
Low risk	1
**4. Is the sample size adequate for establishing relationships (assumption of statistical power)**	
No	0
Yes	1
**5. Measurement**	
How was organizational cynicism measured?	
With a standardized scale	1
Self-labeling without definition of the organizational cynicism concept	0
**6. Measurement**	
How was workplace counterproductive work behavior?	
Self-labeling without definition of the counterproductive work behavior concept	0
With a standardized scale	1
**7. Performance bias: Is there a risk of bias caused by the inadequate measurement of organizational cynicism?**	
High risk	0
Low risk	1
**8. Performance bias: Is there a risk of bias caused by the inadequate measurement of counterproductive work behaviors?**	
High risk	0
Low risk	1
**9. Are the statistical methods appropriate for the study design?**	
No/Can’t tell	0
Yes	1
**10. Were meaningful demographic covariates included?**	
No	0
Yes	1
**11. Were other work factors adjusted for?**	
No	0
Yes	1

### 2.3 Data analysis

CMA 3.0 package program was used for the analysis of the research data. Pearson’s correlation values were used to calculate the effect size of organizational cynicism and counterproductive work behaviors studies. Correlation values were first converted into Fisher’s Z scores and then converted back into Pearson’s correlation values. This process is done as sensitive predictions in effect size calculation.

### 2.4 Evaluating publication bias

In meta-analytic datasets, above a certain level publication bias is examined because it affects the calculated average effect size and causes it to be larger than it should be (Borenstein et al., 2009). Firstly publication bias was analyzed according to Duwal and Tweedie. Accordingly, the normal distribution of the research is slightly left skewed. This left skewness can reach a full normal distribution when 3 more studies are added to the right side of the graph. This result was interpreted as no publication bias in the studies observed between the variables according to Duwal and Tweedie. The funnel plot also supports this assessment. The Classic Fail-Safe N value was also calculated to check for publication bias. For the double-ended Alpha value to exceed 0.05, 9,363 more studies need to be added (*Z* = 40.49; *p* = 0.000). The high number of studies that need to be added indicates reliable studies in terms of publication bias. For this reason, it was accepted that there was no publication bias in the research according to the Classic Fail-Safe N value.

### 2.5 Quality assessment tool

There is no standardized tool for quality assessment of studies in systematic review studies in the field of social sciences. For this reason, the 13-item quality assessment tool used by Nielsen et al. (2016) was adapted and used as 11 items in our study (see Table 1). The researchers performed the quality assessment for each study independently. Then, the quality assessment score given to each study by the researchers was averaged. Thus, it was aimed to make an impartial and objective evaluation in the quality assessment of the studies. The quality assessment results of the studies included in the research are presented in Table 2.

**TABLE 2:** Summary of study characteristics.

Studies number	References	Study title	Quality score	Study type	Publication year	Country	Sample size	Pearson correlation	Scale 1	Scale 2
1.	Ali et al., 2022	“The outcomes of organizational cronyism: a social exchange theory perspective”	9	Article	2022	Pakistan (Asia)	170 (M: US, F: US)	0.577**	Organizational cynicism scale	Counter productive work behavior scale
2.	Ahmed et al., 2024	“The effect of toxic leadership on workplace deviance: the mediating effect of emotional exhaustion, and the moderating effect of organizational cynicism”	9	Article	2024	Egypt (Africa)	243 (M: 74, F: 169)	0.585***	Organizational cynicism scale	Workplace deviance scale
3.	Bal, 2020	“The role of organizational cynicism as a mediator in the relationship between perceived organizational support and counter productive work behavior for public employees”	10	Article	2020	Türkiye (Europa)	419 (M: 229, F: 190)	0.178*	Counterproductive work behavior scale	Organizational cynicism scale
4.	Butt and Yazdani, 2021	“Influence of workplace incivility on counterproductive work behavior: mediating role of emotional exhaustion, organizational cynicism and the moderating role of psychological capital”	7	Article	2021	Pakistan (Asia)	215 (M: 95, F: 120)	0.517**	Counterproductive work behaviors scale	Organizational cynicism scale
5.	Danaeefard and Boustani, 2016	“Injustice perceptions and employees misbehavior in the public organization: exploration of mediating role of employee’s cynicism to organization”	9	Article	2016	Iran (Asia)	420 (M: 256, F: 164)	0.508	Employee’s cynicism scale	Employee’s misbehavior scale
6.	Dar et al., 2020	“Deviant behavior and organizational justice: mediator test for organizational cynicism- the case of Pakistan”	6	Article	2020	Pakistan (Asia)	137 (M: 98, F: 39)	0.504**	Deviant behavior	Organizational cynicism questionnaire
7.	Eren and Demir, 2023	“The effect of perceived pay equity on counterproductive work behaviors: the mediating role of organizational cynicism”	7	Article	2023	Türkiye (Europa)	252 (M: 136, F: 116)	0.365**	Organizational cynicism scale	Counter productive work behavior scale
8.	Evans et al., 2010	“The impact of perceived corporate citizenship on organizational cynicism, ocb, and employee deviance”	6	Article	2011	United States (America)	188 (M: 92, F: 96)	0.38***	Organizational cynicism scale	Employee deviance scale
9.	Evans et al., 2021	“The role of organizational cynicism and conscientiousness in the relationship between ethical leadership and deviance”	7	Article	2021	United States (America)	277 (M: 148, F: 129)	0.25	Organizational cynicism scale	Workplace deviance
10.	Griep et al., 2023	“Perceived identity threat and organizational cynicism in the recursive relationship between psychological contract breach and counterproductive work behavior”	5	Article	2023	Belgium (Europa)	386 (M: 105, F: 281)	0.65***	Counter productive work behavior scale	Organizational cynicism scale
11.	Jiang et al., 2017	“The relationship between authoritarian leadership and employees’ deviant workplace behaviors: the mediating effects of psychological contract violation and organizational cynicism”	9	Article	2017	China (Asia)	391 (M: 271, F: 120)	0.37**	Deviant workplace behaviors questionnaire	Organizational cynicism questionnaire
12.	Kim and Jo, 2024	“An examination of the effects of job insecurity on counterproductive work behavior through organizational cynicism: moderating roles of perceived organizational support and quality of leader-member exchange”	9	Article	2022	South Korea (Asia)	296 (M: 119, F: 177)	0.43**	Organizational cynicism scale	Counterproductive work behavior checklist
13.	Li and Chen, 2018	“The relationship between psychological contract breach and employees’ counterproductive work behaviors: the mediating effect of organizational cynicism and work alienation”	10	Article	2018	China (Asia)	484 (M: 417, F: 77)	0.30***	Counterproductive work behaviors scale	Organizational cynicism questionnaire
14.	Naseer et al., 2021	“When and why organizational cynicism leads to CWBs”	6	Article	2021	Pakistan (Asia)	181 (M: 98, F: 83)	0.38**	Organizational cynicism	Counterproductive work behaviors scale
15.	Nemr and Liu, 2021	“Organizational cynicism as a moderator variable between ethical leadership and counterwork productive behaviors”	10	Article	2021	Egypt (Africa)	400 (M: 244, F: 156)	0.312	Counterproductive work behaviors scale	Organizational cynicism scale
16.	Peng et al., 2021	“Older and less deviant? The paths through emotional labor and organizational cynicism”	9	Article	2021	China (Asia)	681 (M: 276, F: 405)	0.27	Workplace deviance scale	Organizational cynicism scale
17.	Rayan et al., 2018	“Organizational cynicism and counterproductive work behaviors: an empirical study”	9	Article	2018	Egypt (Africa)	327 (M: US, F: US)	0.386**	Counterproductive work behaviors scale	Organizational cynicism scale
18.	Safdar et al., 2022	“Examination of the relationship between organization cynicism and employees workplace behaviors: modeling Islamic (servant) leadership as a moderator”	7	Article	2022	Pakistan (Asia)	280 (M: US, F: US)	0.363**	Organizational cynicism scale	Workplace deviance behavior scale
19.	Saad Saleh Ali and Abdelwahab Ibrahim Elsayed, 2022	“Correlation between organizational cynicism and counterproductive work behaviors among nurses”	9	Article	2022	Egypt (Africa)	550 (M: 135, F: 415)	0.633*	Organizational cynicism scale	Counterproductive behaviors scale
20.	Shahzad and Mahmood, 2012	“The mediating - moderating model of organizational cynicism and workplace deviant behavior: evidence from banking sector in Pakistan”	7	Article	2012	Pakistan (Asia)	332 (M: 210, F: 122)	0.817**	Organizational cynicism scale	Workplace deviant behavior scale
21.	Tong et al., 2020	“The interplay of low identification, psychological detachment, and cynicism for predicting counterproductive work behavior”	6	Article	2020	China (Asia)	382 (M: 162, F: 220)	0.49***	Counterproductive work behaviors scale	Cynicism maslach burnout inventory
22.	Ugwu et al., 2023	“Mediating roles of employee cynicism and workplace ostracism on the relationship between perceived organizational politics and counterproductive work behavior”	10	Article	2023	Nigeria (Africa)	794 (M: US, F: US)	0.232	Organizational cynicism scale	Counterproductive work behaviors scale

*p < 0.05, **p < 0.01, ***p < 0.001.

## 3 Results

Some studies were excluded from the Prisma flow diagram because they applied a different analysis than Pearson’s correlation (Abdi et al., 2016; Afghan et al., 2018; Murad et al., 2021; Sönmez et al., 2024) or one of them were repetitions of a Ph.D. thesis (Bal, 2021).

Table 2 presents the details of the studies included in the research. Accordingly, the studies were conducted in 9 different countries. It is seen that 22 of the studies are research articles. The total number of participants in the studies was 7,331.

### 3.1 Effect size and heterogeneity test

The effect size and heterogeneity test results of the studies conducted with organizational cynicism and counterproductive work behaviors variables according to the random effects model are presented in Table 3.

**TABLE 3:** Effect size and heterogeneity test results.

Variables	Model	n	Total sample	95%						Heterogeneity test			
				Average effect size	*R*	SD	LL	UL	*p*	*Q*	df	*p*	*I* ^2^
OC^1^ and CWBs^2^	Random	22	7,331	0.482	0.448	0.017	0.386	0.578	0.00	382.554	21	0.00	94.511

^1^Organizational cynicism. ^2^Counterproductive work behaviors.

In Table 3, heterogeneity values are calculated as *Q* = 382.554 for 21 degrees of freedom. In the Chi-square table, the cut-off value for 21 degrees of freedom at 0.05 confidence level is 32.621. The calculated *Q*-value is considerably higher than the value in the Chi-square table. Thus, the assumption that the data are heterogeneously distributed is accepted. In addition, according to the I^2^ value, a high heterogeneity of 94.511% was found between the variables. Therefore, the random effects model was used in the research. Cohen’s d effect size classification was used in the interpretation of effect sizes. According to Cohen’s d effect size values between 0.10 and 0.29 refer to a small effect size, between 0.30 and 0.49 refer to a medium effect size, and 0.50 and above refer to a large effect size (Dinçer, 2014). The average effect size of the random effects model is 0.482. The calculated effect size is at medium level and statistically significant (*p* < 0.001). Moreover, there is a positive relationship between organizational cynicism and counterproductive work behaviors *(r* = 0.448). Subgroup analyses were also conducted to explore possible moderators. Three different groups were determined for this purpose. The first one is the sample size. The sample size of the studies was coded in 2 groups (0–250 and 251 +). The effect size of 4 studies with a sample size between 0 and 250 was calculated as 0.502 (SD = 0.122), and the effect size of 18 studies with a sample size of 251 + was calculated as 0.477 (SD = 0.056). No significant differentiation was found between these two groups *(Q* = 0.035; df = 1; *p* = 0.851). Secondly, the continent variable was used. The studies were coded in four different continents (Asia, Europe, Africa and America). The average effect size of 12 studies in Asia was 0.519 (SD = 0.072), the average effect size of 3 studies in Europe was 0.446 (SD = 0.144), the average effect size of 5 studies in Africa was 0.475 (SD = 0.111) and the average effect size of 2 studies in the America was 0.327 (SD = 0.178). After the comparison, no significant difference was found (*Q* = 1.088; df = 2; *p* = 0.78). Finally, the power distance score, one of the dimensions of Hofstede’s Theory of Culture, was used and the countries scoring between 0 and 60 points and the countries scoring 61 + points were coded. The average effect size of the 10 studies with a power distance score between 0 and 60 points was 0.540 (SD = 0.073), and the average effect size of the 12 studies with 61 + points was 0.435 (SD = 0.066). As a result of the comparison no significant difference was found (*Q* = 1.135; df = 1; *p* = 0.287).

**FIGURE 2:**
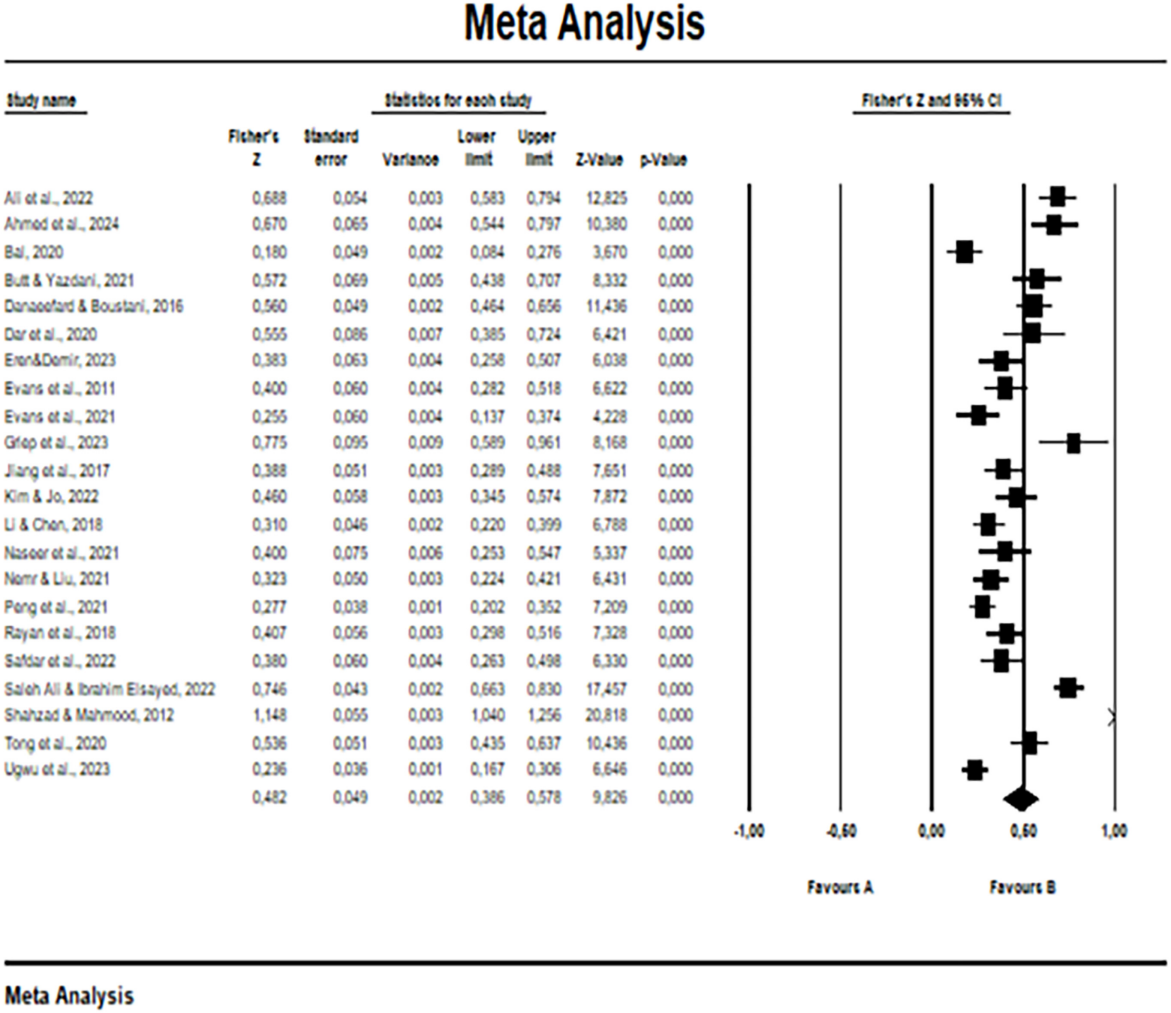
Effect size and 95% CI.

Figure 2 shows the effect size value, standard errors, upper and lower bounds according to 95% confidence intervals and CI values for each study in Fisher’s Z random effects model. The black squares in the figure indicate the estimated effect size of each study. The length of the horizontal lines in the center of the squares indicates the confidence interval of the related studies. A short horizontal line through the center of the squares means that the precision is high and the confidence interval is narrow. Longer lines indicate low precision and wide confidence intervals. The diamond symbol at the bottom of the figure indicates the overall effect size and confidence interval. The width of the diamond indicates the confidence interval of the effect size and the height of the diamond indicates the risk ratio or odds ratio (Benligül et al., 2022).

## 4 Discussion

As a result of the research, it was determined that there is a significant, positive relationship between organizational cynicism and counterproductive work behaviors and a medium effect size. This result is in parallel with the literature (Ahmed et al., 2013; Mathur et al., 2013; Abdi et al., 2016; Hussain and Malik, 2019; Oliveira et al., 2020; Sen et al., 2022; Loh and Azalea, 2023). Social exchange theory argues that interpersonal relationships are based on mutual norms. Therefore, considering that both organizational cynicism and counterproductive work behaviors are based on employee-manager, employee-employee or employee-customer relationships in organizations, it can be argued that these relationships are incompatible or unfair. Especially as the perception of organizational injustice increases, cynical attitudes and behaviors may emerge in employees toward the organization. Similarly, as stated in Adams and Freedman (1976) equity theory, employees’ perception of injustice arises when the output they receive in return for their labor is less than their labor. To compensate, employees may deliberately slow down, miss work, be absent, or take longer rest and meal breaks. These behaviors of the employees fit the minor behaviors in Robinson and Bennett’s (1995) binary taxonomic classification of counterproductive work behaviors. Therefore, it can be said that the research results supports both the social exchange theory and Adams and Freedman’s equity theory. The job demands resources model emphasizes that high levels of job demand from employees play a stressor role and cause tension, thus making employees more cynical and this continues as a cycle. Although job resources play a motivating role on employees, high job demands can create pressure on employees. Stress and pressure on employees can lead them to dysfunctional behaviors or revenge-type behaviors. Organizations can manage the high job demands on their employees through fair and equal workload practices, flexible working hours, and encouraging greater participation in work-related decisions.

Employees’ perception of fairness plays an important role in the emergence of organizational cynicism and counterproductive work behaviors. However, inter-employee relations, attitudes and behaviors of leaders or managers, organizational support and organizational culture may also be other determining factors. Leadership behaviors and organizational climate in the organization should also be taken into consideration in the formation of organizational cynicism and counterproductive work behaviors (Dobbs, 2014; Polatcan and Titrek, 2014; Puni et al., 2016; Huang et al., 2021; Murad et al., 2021). Moreover, toxic organizational structure, lack of organizational support, inconsistent organizational practices, and workplace dynamics can also lead to cynical feelings toward the organization and counterproductive work behaviors among employees (Eigenstetter et al., 2007; Adıgüzel and Okçu, 2021; Kim, 2021). Additionally, the decreased self-esteem of cynical individuals and their increased sensitivity to psychological contract breaches may encourage them to engage in counterproductive work behaviors (Ahmed and Zhang, 2024). Like the snowball mechanism, the perception of psychological contract breach for cynical individuals may play a triggering role for counterproductive work behaviors (Griep and Vantilborgh (2018). All these results and the research reveal that there is a significant relationship between organizational cynicism and counterproductive work behaviors. It shows that factors such as psychological contract breach, workplace incivility, discrimination, exclusion and perception of injustice may also play a role in the relationship between organizational cynicism and counterproductive work behaviors. Furthermore, firstly organizational justice, and organizational climate, organizational support, organizational culture, organizational politics and leadership styles are important and effective factors. The findings of the research indicate that cynical employees in organizations may engage in counterproductive work behaviors. Counterproductive work behaviors pose a threat to organizations, other employees and customers. For this reason, managers and leaders can take measures against the factors that reveal cynicism in their employees by considering organizational dynamics. For this purpose, managers can communicate directly with their employees by implementing an open-door policy, listening to employees’ opinions and suggestions, making ethical, egalitarian and fair decisions, and promoting organizational welfare.

## 5 Conclusion

The research found that there is a significant and positive relationship between organizational cynicism and counterproductive work behaviors, and the effect size is at medium level. In this relationship, it can be said that as employees’ perception of organizational cynicism increases, they tend to engage in counterproductive work behaviors more like a cycle. Organizational cynicism is generally associated with cynical attitudes of employees toward the organization. Counterproductive work behaviors can be a result of organizational cynicism, workplace incivility or psychological contract violation, as well as factors related to individual or interpersonal relationships. In this sense, organizational cynicism is one of the important factors that trigger counterproductive work behaviors for employees. Risk factors for the emergence of both cynicism and counterproductive work behaviors in organizations may include perceptions of organizational injustice, organizational culture, organizational climate, experiencing negative emotions, burnout, workplace incivility, work stress, high job demands, exclusion, unethical leadership styles, and unsupportive toxic organizational structures (Kwak, 2016; Zaghini et al., 2016; Jiang et al., 2017; De Clercq et al., 2019; Aydın Küçük, 2019; Naseer et al., 2021; Prajogo et al., 2020; Tutar et al., 2021; Saifullah and Javed, 2023).

### 5.1 Implications for the theory and practice

The two main antecedents of organizational cynicism are personality and organizational factors. Personality characteristics include gender, value judgments, habits, attitudes and education level. Organizational factors include changes at work, organizational justice, organizational support, organizational policies and practices, and relationships with leaders (Chiaburu et al., 2013; Evans et al., 2021; Polatcan and Titrek, 2014; Brandes, 1997, James, 2005; Kim et al., 2019). And it can be said that all these antecedents are factors that affect employees’ productivity, job satisfaction and organizational commitment (Khan et al., 2016; Yaşar and Özdemir, 2016; Wicaksono and Muafi, 2021). Antecedents of counterproductive work behaviors generally stand out as individual and situational factors (Spector, 2011; Oliveira et al., 2020; Yang and Diefendorff, 2009; Bolton et al., 2010; Scott and Judge, 2013). It can be argued that the emergence of organizational cynicism theoretically constitutes one of the reasons for counterproductive work behaviors in organizations. Job demands (time pressure, conflicts, excessive labor inspection) may cause stress, anxiety, tension and burnout over time. These can lead to negative feelings toward work and organization, cynical attitudes and counterproductive work behaviors. Similarly, when employees believe that the organization and managers do not treat them fairly and that organizational resources are not allocated equally and fairly, they may engage in counterproductive work behaviors to ensure equality and fairness. The results of this research reveal that organizational cynicism and counterproductive work behaviors are important problems for both organizations and employees in today’s working life and may be a problem in the future. Therefore, these are important factors that can be effective in decreasing the productivity and performance of organizations and employees. Regarding the practical implications of the research, it can be suggested that leaders and managers should improve communication and transparency in order to reduce organizational cynicism and counterproductive work behaviors. In addition, it can be said that creating a healthy, supportive, fair and ethical organizational climate in organizations is also very important. Furthermore, it can be said that organizations should show equal, fair attitudes and behaviors to employees, create opportunities for employees to gain autonomy over work when necessary, and provide social support among employees. Today’s organizational structures are composed of employees from different cultures. Organizations should consider that these differences may lead to differences in employees’ thoughts, feelings, behaviors, attitudes and values. This research, which synthesizes studies conducted in 4 continents and 9 countries, reveals a generalizable result between organizational cynicism and counterproductive work behaviors. Organizations can take various measures to reduce organizational cynicism. For this purpose, a participatory and fair organizational culture can be created. The organization should be open and honest in communicating with employees, this can prevent employees’ negative feelings and attitudes toward the organization. To control anger and stress, which underlie organizational cynicism and counterproductive work behaviors, organizations should provide regular stress and anger prevention and management training to their employees. Employees should be treated fairly, because counterproductive work behaviors and organizational cynicism are often a reaction to employees’ unfair attitude toward organizational justice. Especially managers and Human Resources (HR) specialists who develop organizational policies are influential on employees in this process. Managers can directly affect their employees’ working environment and indirectly affect their well-being and job performance (Bakker and Demerouti, 2016). Managers should take care to distribute resources such as promotions and rewards equally and transparently according to performance. Employees should be included in decision-making processes Managers should give importance to respect and human approach in their communication with employees. While addressing the main causes of organizational cynicism, HR professionals can implement various strategies to reduce counterproductive work behaviors. These are: 1. Providing honest and timely information to employees. 2. Providing realistic information to employees during recruitment and orientation processes, 3. Developing an HR policy that is sensitive to psychological contract breaches, 4. Establishing employee support programs (such as psychological counseling and stress management), 5. Following a zero-tolerance policy for unethical behavior. In line with these results, organizations should take into account that every employee is affected by negative attitudes, situations, policies, practices and conditions. Therefore, it should be considered that fair, supportive, developmental, transparent, democratic attitudes and organizational practices, ethical and democratic leadership styles will create a healthy organizational structure. If these can be meticulously realized in organizations, employees’ commitment to the organization, job performance and trust in the organization can be positively affected.

### 5.2 Limitations and future directions

In line with the inclusion criteria, the study data covers the period between 2010 and 2024. Therefore, the study is limited to the period examined, the studies accessed, the databases, the articles written in English, the method used in these studies (Pearson correlation analysis) and the accessibility of the journals (electronic). In this study, meta-analysis was conducted on correlation values, so deterministic results in correlational studies are limited. Therefore, future studies may include experimental and longitudinal research. Potential areas for further research: Sector-specific differences in the relationship between organizational cynicism and counterproductive work behaviors, possible mediating variables (workplace incivility, nepotism, psychological contract breach, job insecurity, ethical leadership, organizational climate) can be examined. In addition, specific variables that may have an impact on organizational cynicism and counterproductive work behaviors can be examined through meta-analysis. In addition, the effects of flexible work arrangements (such as part-time working, online working, compressed work week) and leadership styles on organizational cynicism and counterproductive work behaviors can also be investigated.

## Data Availability

The datasets presented in this study can be found in online repositories. The names of the repository/repositories and accession number(s) can be found at: https://drive.google.com/dri ve/folders/1b8AVSncGmPC0Ajbkb5NTdJoAicIY-HOm?usp=drive _link.
