# Supplementary material for: Investigation of the relationship between organizational cynicism and counterproductive work behaviors: a systematic review and meta-analysis
Source: Front Psychol. 2025 Nov 26;16:1529798. doi: 10.3389/fpsyg.2025.1529798 (PMC12689420; doi:10.3389/fpsyg.2025.1529798)
Supplement: Supplementary file 1 [file Supplementary_file_1.docx]

| **Section and Topic** | **Item #** | **Checklist item** | **Location where item is reported** |
| --- | --- | --- | --- |
| **TITLE** | | |  |
| Title | 1 | Identify the report as a systematic review. | Title |
| **ABSTRACT** | | |  |
| Abstract | 2 | See the PRISMA 2020 for Abstracts checklist. | Abstract title |
| **INTRODUCTION** | | |  |
| Rationale | 3 | Describe the rationale for the review in the context of existing knowledge. | İntroduction title |
| Objectives | 4 | Provide an explicit statement of the objective(s) or question(s) the review addresses. | İntroduction and at the end of the counterproductive work behaviours title,before the methods title |
| **METHODS** | | |  |
| Eligibility criteria | 5 | Specify the inclusion and exclusion criteria for the review and how studies were grouped for the syntheses. | Eligibility criteria title |
| Information sources | 6 | Specify all databases, registers, websites, organisations, reference lists and other sources searched or consulted to identify studies. Specify the date when each source was last searched or consulted. | Materials and Methods title |
| Search strategy | 7 | Present the full search strategies for all databases, registers and websites, including any filters and limits used. | Materials and Methods title |
| Selection process | 8 | Specify the methods used to decide whether a study met the inclusion criteria of the review, including how many reviewers screened each record and each report retrieved, whether they worked independently, and if applicable, details of automation tools used in the process. | Eligibility criteria title |
| Data collection process | 9 | Specify the methods used to collect data from reports, including how many reviewers collected data from each report, whether they worked independently, any processes for obtaining or confirming data from study investigators, and if applicable, details of automation tools used in the process. | Materials and Methods, selection and coding variables titles |
| Data items | 10a | List and define all outcomes for which data were sought. Specify whether all results that were compatible with each outcome domain in each study were sought (e.g. for all measures, time points, analyses), and if not, the methods used to decide which results to collect. | Table 1 under the selection and coding variables title |
|  | 10b | List and define all other variables for which data were sought (e.g. participant and intervention characteristics, funding sources). Describe any assumptions made about any missing or unclear information. | Table 1 under the selection and coding variables title and under the methods title |
| Study risk of bias assessment | 11 | Specify the methods used to assess risk of bias in the included studies, including details of the tool(s) used, how many reviewers assessed each study and whether they worked independently, and if applicable, details of automation tools used in the process. | Evaluating publication bias title and under the selection and coding variables title |
| Effect measures | 12 | Specify for each outcome the effect measure(s) (e.g. risk ratio, mean difference) used in the synthesis or presentation of results. | Table 2: effect size and heterogeneity test results under the results title |
| Synthesis methods | 13a | Describe the processes used to decide which studies were eligible for each synthesis (e.g. tabulating the study intervention characteristics and comparing against the planned groups for each synthesis (item #5)). | Selection and coding variables title |
|  | 13b | Describe any methods required to prepare the data for presentation or synthesis, such as handling of missing summary statistics, or data conversions. | Materials and Methods title |
|  | 13c | Describe any methods used to tabulate or visually display results of individual studies and syntheses. | Selection and coding variables title |
|  | 13d | Describe any methods used to synthesize results and provide a rationale for the choice(s). If meta-analysis was performed, describe the model(s), method(s) to identify the presence and extent of statistical heterogeneity, and software package(s) used. | Materials and Methods, data analysis title |
|  | 13e | Describe any methods used to explore possible causes of heterogeneity among study results (e.g. subgroup analysis, meta-regression). | Results and evaluating publication bias title |
|  | 13f | Describe any sensitivity analyses conducted to assess robustness of the synthesized results. | Evaluating publication bias title |
| Reporting bias assessment | 14 | Describe any methods used to assess risk of bias due to missing results in a synthesis (arising from reporting biases). | Evaluating publication bias title (Classic fail-safe N and Kendall) |
| Certainty assessment | 15 | Describe any methods used to assess certainty (or confidence) in the body of evidence for an outcome. | Materials and Methods, data analysis |
| **RESULTS** | | |  |
| Study selection | 16a | Describe the results of the search and selection process, from the number of records identified in the search to the number of studies included in the review, ideally using a flow diagram. | Materials and Methods (Figure 1. Prisma flow diagram) |
|  | 16b | Cite studies that might appear to meet the inclusion criteria, but which were excluded, and explain why they were excluded. | Evaluating publication bias title (explanation under the Figure 1) |
| Study characteristics | 17 | Cite each included study and present its characteristics. | Table 1 under the selection and coding variables title and reference lists |
| Risk of bias in studies | 18 | Present assessments of risk of bias for each included study. | Evaluating publication bias title |
| Results of individual studies | 19 | For all outcomes, present, for each study: (a) summary statistics for each group (where appropriate) and (b) an effect estimate and its precision (e.g. confidence/credible interval), ideally using structured tables or plots. | Results (Figure 2. Effect size and forest plots of the studies) |
| Results of syntheses | 20a | For each synthesis, briefly summarise the characteristics and risk of bias among contributing studies. | Table 1 under the selection and coding variables and evaluating publication bias title |
|  | 20b | Present results of all statistical syntheses conducted. If meta-analysis was done, present for each the summary estimate and its precision (e.g. confidence/credible interval) and measures of statistical heterogeneity. If comparing groups, describe the direction of the effect. | Results title(Table 2: effect size and heterogeneity test results and Figure 2. Effect size and forest plots of the studies) |
|  | 20c | Present results of all investigations of possible causes of heterogeneity among study results. | Results title(Table 2: effect size and heterogeneity test results) |
|  | 20d | Present results of all sensitivity analyses conducted to assess the robustness of the synthesized results. | Results (Figure 2. Effect size and forest plots of the studies) |
| Reporting biases | 21 | Present assessments of risk of bias due to missing results (arising from reporting biases) for each synthesis assessed. | Selection and coding variables title |
| Certainty of evidence | 22 | Present assessments of certainty (or confidence) in the body of evidence for each outcome assessed. | Results title(Table 2: effect size and heterogeneity test results and Figure 2. Effect size and forest plots of the studies) |
| **DISCUSSION** | | |  |
| Discussion | 23a | Provide a general interpretation of the results in the context of other evidence. | Discussion title |
|  | 23b | Discuss any limitations of the evidence included in the review. | Limitations and suggestions title |
|  | 23c | Discuss any limitations of the review processes used. | Limitations and suggestions title |
|  | 23d | Discuss implications of the results for practice, policy, and future research. | Limitations and suggestions title |
| **OTHER INFORMATION** | | |  |
| Registration and protocol | 24a | Provide registration information for the review, including register name and registration number, or state that the review was not registered. | Conflict of interest |
|  | 24b | Indicate where the review protocol can be accessed, or state that a protocol was not prepared. | Conflict of interest ( a link to the data used for all analyses and data from the included studies are provided) |
|  | 24c | Describe and explain any amendments to information provided at registration or in the protocol. | Selection and coding variables title |
| Support | 25 | Describe sources of financial or non-financial support for the review, and the role of the funders or sponsors in the review. | Supporting information title |
| Competing interests | 26 | Declare any competing interests of review authors. | Conflict of interest title |
| Availability of data, code and other materials | 27 | Report which of the following are publicly available and where they can be found: template data collection forms; data extracted from included studies; data used for all analyses; analytic code; any other materials used in the review. | Data set and analysis results |

*From:*  Page MJ, McKenzie JE, Bossuyt PM, Boutron I, Hoffmann TC, Mulrow CD, et al. The PRISMA 2020 statement: an updated guideline for reporting systematic reviews. BMJ 2021;372:n71. doi: 10.1136/bmj.n71
