# Supplementary figures and images for: Investigation of the relationship between organizational cynicism and counterproductive work behaviors: a systematic review and meta-analysis
Source: Front Psychol. 2025 Nov 26;16:1529798. doi: 10.3389/fpsyg.2025.1529798 (PMC12689420; doi:10.3389/fpsyg.2025.1529798)

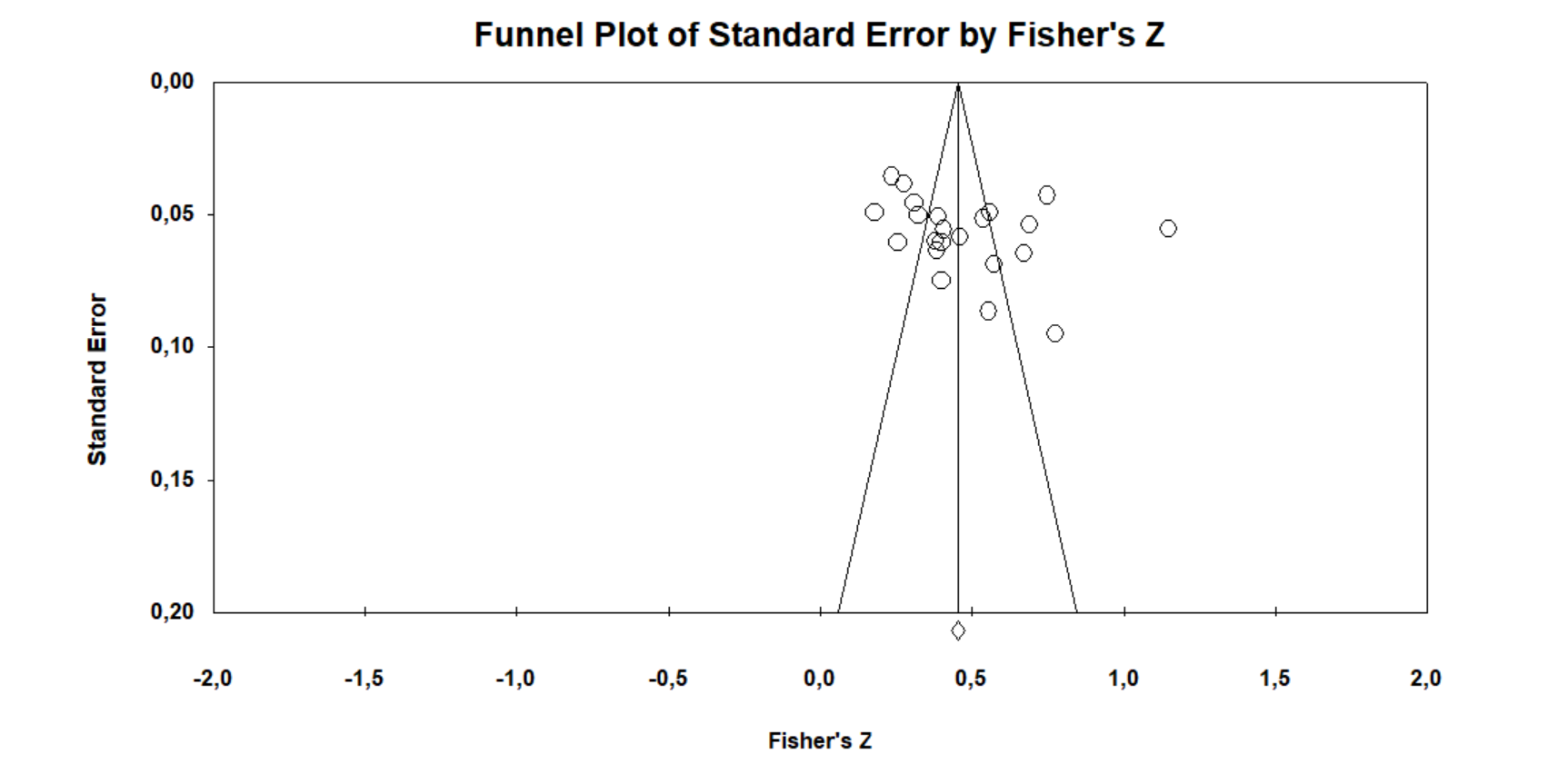

Supplement: Supplementary file 2 [file Image_1.PNG]
